# The chloroplast genome of *Fallopia aubertii* (Polygonaceae) from Xining, China

**DOI:** 10.1080/23802359.2021.2001390

**Published:** 2021-11-23

**Authors:** Yongchang Lu, Wancui Wang, Yingxiang Yu, Qian Ma, Huan Wang

**Affiliations:** a Collage of Pharmacy, Qinghai Minzu University, Xining, China; b Key Laboratory for Tibet Plateau Phytochemistry of Qinghai Province, Xining, China

**Keywords:** Chloroplast genome, evolutionary analysis, *Fallopia aubertii*, Polygonaceae

## Abstract

*Fallopia aubertii* (L.Henry) Holub (Polygonaceae), a woody plant with a voluble stem, is used as a folk herbal medicines for the treatment of gout, fever and pneumonia. To better understand the molecular genetics of *F. aubertii*, its complete chloroplast genome was sequenced and annotated. The assembled chloroplast genome is a circular 160,951 bp sequence consisting of large single copy (87,279 bp) and small single copy (13,394 bp) regions, separated by two inverted repeat regions (30,860 bp each). The genome contains 131 genes including 86 protein-coding, 37 tRNA and 8 tRNA genes. Phylogenetic analysis based on the complete chloroplast genome showed that *F. aubertii* is more closely related to *M. australis* than to *F. sachalinensis*, which exhibited a polyphyletic relationship with respect to *F. aubertii*. These results require further analyses. This study provides additional data for reconstructing species relationships in *Fallopia*.

*Fallopia aubertii* (L. Henry) Holub (Polygonaceae) is classified in the tribe Polygoneae (Polygonaceae: Polygonoideae) and is a woody plant with a voluble stem (Schuster et al. 2011). This species is used in folk herbal medicines for the treatment of gout, fever and pneumonia (Wang et al. 2019). Due to fewer recombination incidents, lower nucleotide replacement rates, and the typical maternal inheritance (Wolfe et al. 1987), the chloroplast genome has been widely employed to decipher phylogenetic relationships among species (Moore et al. 2010; Shaw et al. 2014; Zhu et al. 2021). In the present study, the complete chloroplast genome of *F*. *aubertii* was *de novo* assembled using Illumina reads and the features of this chloroplast genome were fully elucidated. Then, a phylogenetic analysis of *F. aubertii* and its allies was carried out.

The fresh young leaves of *F. aubertii* were gathered from the campus of Qinghai Minzu University in Xining Qinghai Province of China (36.59°N, 101.82°E) and authenticated by professor Yongchang Lu, one of the authors of this paper. The voucher specimen (specimen accession number: LYC2020001) was deposited in the Qinghai-Tibetan Plateau Museum of Biology, Chinese Academy of Sciences (QTPMB; http://www.nwipb.cas.cn/znbm/qzgyswbbg/bbgjj/, Shilong Chen, herbarium@nwipb.cas.cn). Total DNA was extracted from the fresh leaves with the Plant Genomic DNA Kit (DP305, TIANGEN Biotech Co., Ltd., Beijing, China). The genome sequencing was performed on an Illumina HiSeq Platform (Illumina, San Diego, CA) by Genepioneer Biotechnologies Inc., Nanjing, China. Approximately 4.33 GB of clean data were generated. The sequencing reads were mapped to the reference chloroplast genomes (GenBank accession number: MK842154.1; MN202599.1; MN202598.1; NC_037705.1) using the Bowtie2 software (Langmead and Salzberg, 2012). The SPAdes version 3.10.1 (Bankevich et al. 2012) and SSPACE version 2.0 (Boetzer et al. 2011) were used to assemble the chloroplast genome using default settings. The chloroplast genome was annotated with CPGAVAS2 (http://www.herbalgenomics.org/cpgavas2) (Shi et al. 2019) and the sequence coordinates for the genes were verified by BLAST search against the *F. sachalinensis* (GenBank accession number: MK842154.1) chloroplast genome.

A total of 32 complete plastome sequences of *F. aubertii* and its allies were aligned by MAFFT version 7.473 (Katoh and Standley 2013) using default settings. The evolutionary history of the sequences was inferred using the maximum-likelihood optimality criterion based on the Tamura–Nei model (Tamura and Nei 1993), designating *Afrobrunnichia erecta* as the out-group. The tree with the highest log likelihood (-421955.08) is depicted. The percentage of trees in which the associated taxa clustered together is shown next to the branches. Initial tree(s) for the heuristic search were obtained automatically by applying neighbor-joining and BioNJ algorithms to a matrix of pairwise distances estimated using the maximum composite likelihood approach, and then selecting the topology with superior log likelihood value. The tree was drawn to scale, with branch lengths measured in the number of substitutions per site. The analysis involved 32 nucleotide sequences. All positions containing gaps and missing data were eliminated. There was a total of 131,503 positions in the final dataset. Evolutionary analyses were conducted in MEGA7 (Kumar et al. 2016).

The complete chloroplast genome of *F. aubertii* is 160,951 bp, consisting of large single copy (LSC; 87,279 bp) and small single copy (SSC; 13,394 bp) regions, separated by a pair of inverted repeat regions (IRa and IRb; 30,860 bp each). The genome contains 131 genes including 86 protein-coding, 37 transfer RNAs and 8 ribosomal RNAs. The GC content of this genome is 37.59%. The protein-coding genes, transfer RNAs, and ribosomal RNAs account for 65.65%, 28.24%, and 6.11% of all annotated genes, respectively.

The phylogenetic analysis showed that *F*. *aubertii* and *Muehlenbeckia australis* were closely related (Figure 1). This result is similar to the findings of Schuster et al. (2011). That study indicated that *Fallopia* and *Muehlenbeckia* were closely related. However, *F. Aubertii* was not included in the Schuster et al. study, nor was it in their subsequent investigations (Schuster et al. 2015, 2018). The result that *F. aubertii* is more closely related to *M. australis* than to *F. sachalinensis*, which exhibited a polyphyletic relationship with respect to *F. aubertii*. These results require further analyses. This study provides additional data for reconstructing species relationships in *Fallopia*.

**Figure 1.**
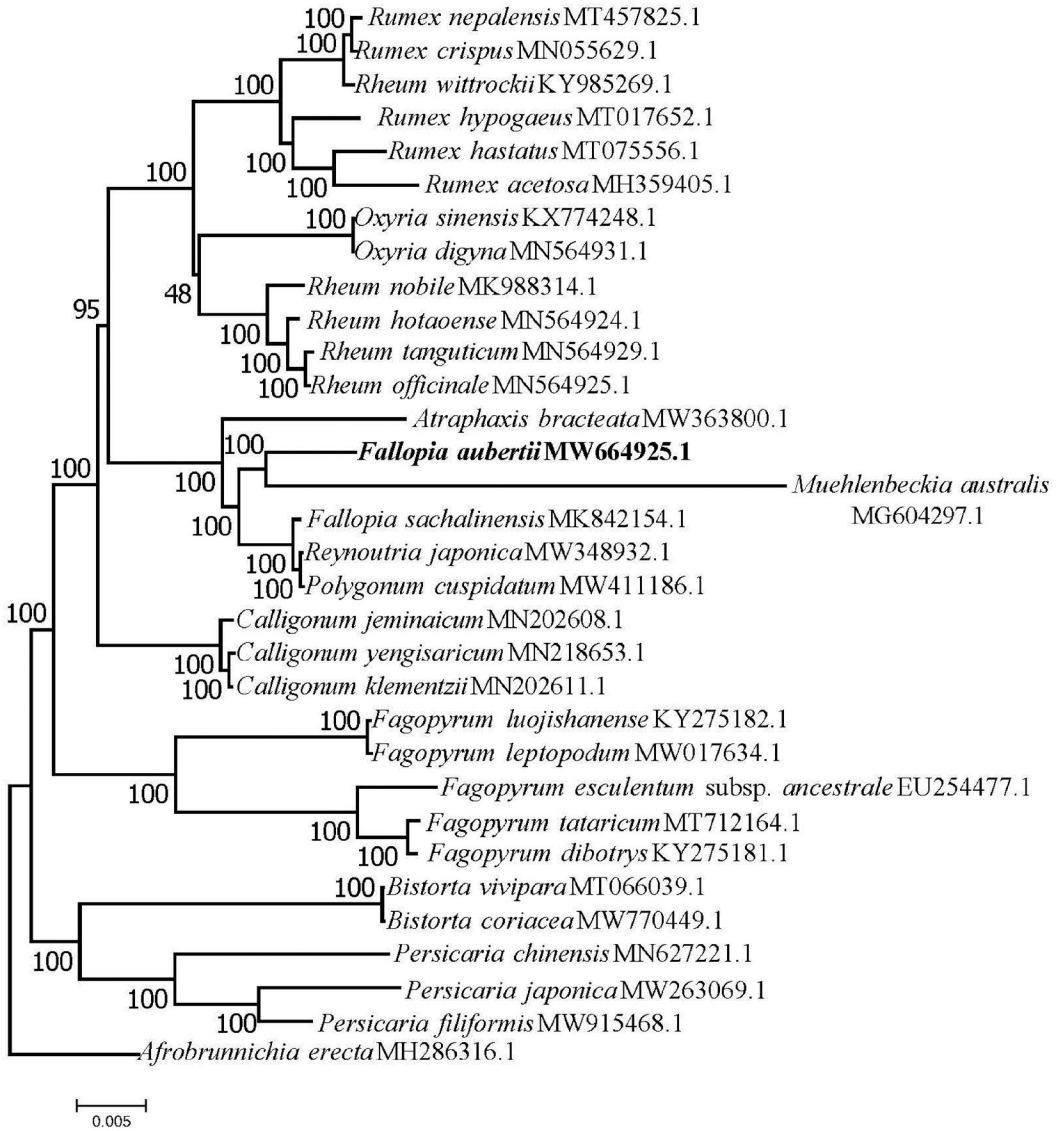
Maximum-likelihood (ML) tree of 32 species based on the complete chloroplast sequences. Numbers above branches are bootstrap percentages (based on 200 replicates).

## Data Availability

The genome sequence data obtained in this study is openly available in GenBank of NCBI at https://www.ncbi.nlm.nih.gov/ under the accession number MW664925. The associated BioProject, SRA, and Bio-Sample numbers are PRJNA728795, SRR14514373, and SAMN19115186, respectively.
